# PorZ, an Essential Component of the Type IX Secretion System of *Porphyromonas gingivalis*, Delivers Anionic Lipopolysaccharide to the PorU Sortase for Transpeptidase Processing of T9SS Cargo Proteins

**DOI:** 10.1128/mBio.02262-20

**Published:** 2021-02-23

**Authors:** Mariusz Madej, Zuzanna Nowakowska, Miroslaw Ksiazek, Anna M. Lasica, Danuta Mizgalska, Magdalena Nowak, Anna Jacula, Monika Bzowska, Carsten Scavenius, Jan J. Enghild, Joseph Aduse-Opoku, Michael A. Curtis, F. Xavier Gomis-Rüth, Jan Potempa

**Affiliations:** a Department of Microbiology, Faculty of Biochemistry, Biophysics, and Biotechnology, Jagiellonian University, Krakow, Poland; b Department of Bacterial Genetics, Institute of Microbiology, Faculty of Biology, University of Warsaw, Warsaw, Poland; c Department of Oral Immunology and Infectious Diseases, University of Louisville School of Dentistry, Louisville, Kentucky, USA; d Department of Cell Biochemistry, Faculty of Biochemistry, Biophysics, and Biotechnology, Jagiellonian University, Krakow, Poland; e Interdisciplinary Nanoscience Center (iNANO) and Department of Molecular Biology, Aarhus University, Åarhus, Denmark; f Centre for Host-Microbiome Interactions, Faculty of Dentistry, Oral and Craniofacial Sciences, King’s College London, London, United Kingdom; g Proteolysis Lab, Department of Structural Biology, Molecular Biology Institute of Barcelona, Higher Scientific Research Council (CSIC), Barcelona, Catalonia, Spain; Georgia Institute of Technology School of Biological Sciences

**Keywords:** *Porphyromonas gingivalis*, T9SS, gingipains, lipopolysaccharide, secretion

## Abstract

Cargo proteins of the type IX secretion system (T9SS) in human pathogens from the Bacteroidetes phylum invariably possess a conserved C-terminal domain (CTD) that functions as a signal for outer membrane (OM) translocation. In Porphyromonas gingivalis, the CTD of cargos is cleaved off after translocation, and anionic lipopolysaccharide (A-LPS) is attached. This transpeptidase reaction anchors secreted proteins to the OM. PorZ, a cell surface-associated protein, is an essential component of the T9SS whose function was previously unknown. We recently solved the crystal structure of PorZ and found that it consists of two β-propeller moieties, followed by a CTD. In this study, we performed structure-based modeling, suggesting that PorZ is a carbohydrate-binding protein. Indeed, we found that recombinant PorZ specifically binds A-LPS *in vitro*. Binding was blocked by monoclonal antibodies that specifically react with a phosphorylated branched mannan in the anionic polysaccharide (A-PS) component of A-LPS, but not with the core oligosaccharide or the lipid A endotoxin. Examination of A-LPS derived from a cohort of mutants producing various truncations of A-PS confirmed that the phosphorylated branched mannan is indeed the PorZ ligand. Moreover, purified recombinant PorZ interacted with the PorU sortase in an A-LPS-dependent manner. This interaction on the cell surface is crucial for the function of the “attachment complex” composed of PorU, PorZ, and the integral OM β-barrel proteins PorV and PorQ, which is involved in posttranslational modification and retention of T9SS cargos on the bacterial surface.

## INTRODUCTION

Periodontal disease is one of the most prevalent infection-driven chronic inflammatory diseases in humans (1). The disease is the result of a host immune response against dysbiotic microorganisms accumulated in the gingival crevice that results in destruction of tooth-supporting tissues. If left untreated, up to 15% of affected adults develop severe periodontitis, which may result in tooth loss (2). In addition, periodontal infection is associated with systemic disorders such as Alzheimer’s disease, osteoporosis, diabetes, rheumatoid arthritis, and respiratory and cardiovascular diseases (3–6).

**FIG 1:**
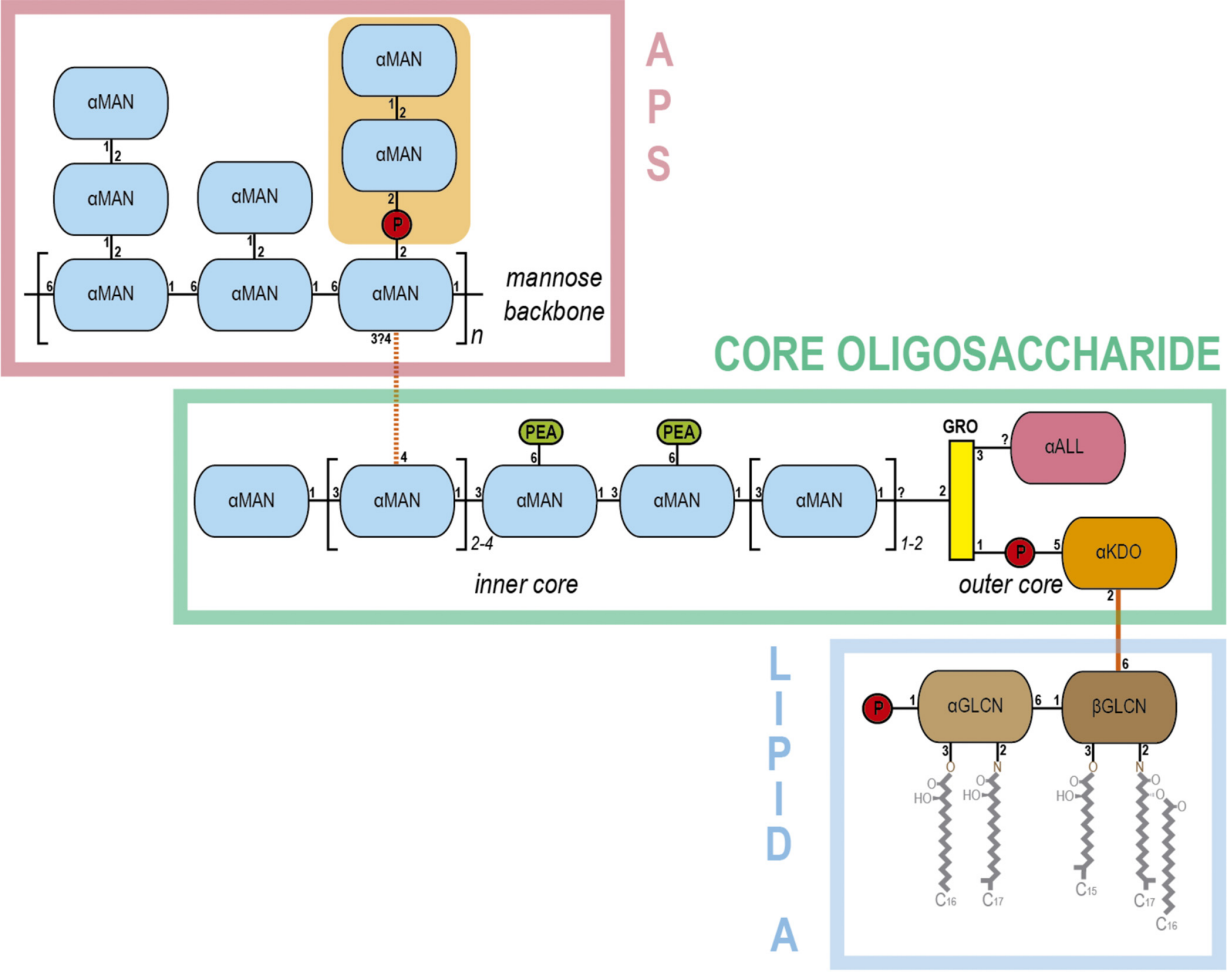
Anionic lipopolysaccharide (A-LPS) of P. gingivalis. A scheme depicting the modular structure of the anionic lipopolysaccharide (A-LPS) of P. gingivalis is shown. A-LPS consists of the A-type anionic distal polysaccharide (APS), the core oligosaccharide, and the endotoxin or lipid A. APS is made of an α-d-mannose (αMAN) backbone, which has side chains of one or two αMANs. One of these side chains contains the branched phosphomannan element recognized by PorZ (orange background). The core oligosaccharide spans a linear chain of αMANs that constitute the inner core. One of the monomers is linked to the mannose backbone of APS (orange dashed line). The two central αMANs of the inner core are linked to phosphoethanolamine (PEA). The inner core chain of αMANs is linked to the outer core through a glycerol molecule (GRO). The latter is linked to α-d-allosamine (αALL) and to a 3-deoxy-d-α-manno-oct-2-ulopyranosonic acid (αKDO) via a phosphate. Monomer αKDO is linked (orange line) to a β-d-glucosamine (βGLCN) from lipid A. The latter is linked to a α-d-glucosamine (αGLCN) and gives rise to the disaccharide bone structure of lipid A, which carries a phosphate and the lipids. Phosphate groups are depicted as red circles. The Δ*pg1142* mutant lacks an O-antigen polymerase and its A-LPS is composed of the single APS unit. The Δ*pg0129* mutant, in turn, affects an α-1,3-mannosyltransferase, which leads to a truncated core that lacks α-(1→3)-linked mannoses and is devoid of APS (39).

The Gram-negative anaerobic bacterium Porphyromonas gingivalis is a major periodontal pathogen that resides in the human gingival crevice. As an asaccharolytic organism, it acquires nutrients in the form of peptides from proteins localized in the gingival crevicular fluid (7, 8). In contrast to the majority of Gram-negative bacteria, P. gingivalis produces two different types of lipopolysaccharide (LPS) molecules (9–11). Both contain the conserved lipid A endotoxin and a core oligosaccharide, but they differ in the highly variable polysaccharide (PS), called the O-antigen, attached to the core. The PS of the more common O-LPS (O-PS) consists of repeating units of a single tetrasaccharide, whereas anionic polysaccharide (A-LPS) consists of a phosphorylated branched mannan (9–11) (Fig. 1).

To thrive in the inflammatory environment of its colonization site (12), P. gingivalis secretes an array of proteinaceous virulence factors. These proteins, including the major extracellular proteolytic agents RgpA, RgpB, and Kgp (collectively called gingipains) (13, 14), possess an N-terminal signal peptide that directs their translocation through the inner membrane (IM) into the periplasmic space via the *Sec* system. In addition, they have a conserved C-terminal domain (CTD) with an Ig-like fold involved in targeted transport across the outer membrane (OM) via the type IX secretion system (T9SS) (15–19).

Despite participating only in OM translocation, the T9SS spans both the IM and the OM (20–22). Its protein components reside on the IM (PorL and PorM) (23, 24) or in the periplasm (PorN, PorK, PorW, and PorE) (25–27); constitute the OM translocon itself (Sov, PorV, PIP, and Plug) (28); or are anchored to the cell surface (PorZ and PorU) via interactions with integral OM β-barrel proteins (PorV and PorQ, respectively) (29). After translocation through the OM, the surface-located PorV-anchored sortase PorU cleaves the CTD and attaches an anionic lipopolysaccharide (A-LPS) molecule, thereby fastening secreted proteins to the OM (30). Expression of the major T9SS components is regulated by PorX and PorY, which form a unique two-component system (31).

Inactivation of any essential component of the T9SS, including PorZ (32), leads to lack of pigmentation in the mutant strain and accumulation of unprocessed proteins, e.g., inactive progingipains, in the periplasm. In this study, we found that in addition to progingipains, the Δ*porZ* deletion mutant also accumulates A-LPS in the periplasmic space, as previously described for the *porV* (Δ*porV*), *porT* (Δ*porT*), and *porU* (Δ*porU*) deletion mutants (33). Furthermore, we showed that PorZ interacts with A-LPS through the phosphorylated branched mannan (Manα1-2Manα1-phosphate) of the A-PS. Finally, we found that A-LPS promotes the interaction between PorZ and the PorU sortase, supporting the idea that, together with PorV and PorQ, PorZ and PorU form a large “attachment complex” engaged in modifying and anchoring T9SS cargos to the P. gingivalis surface (29).

## RESULTS

### A-LPS accumulates in the periplasm of the Δ*porZ* mutant.

**FIG 2:**
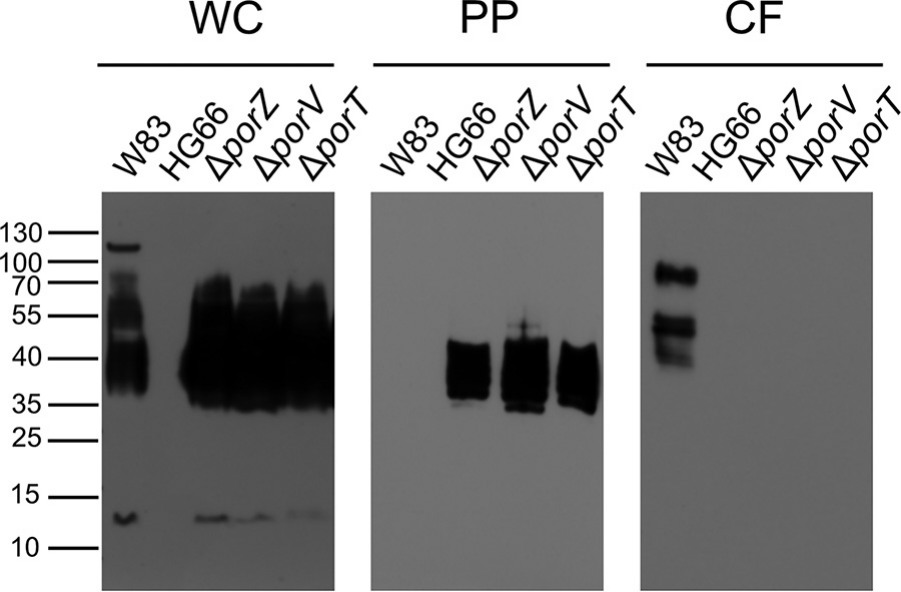
Western-blot analysis to detect A-LPS in subcellular fractions of P. gingivalis cultures. P. gingivalis cultures of W83, HG66, Δ*porZ*, Δ*porV*, and Δ*porT* strains were fractionated as described in Materials and Methods. Washed cells (WC), periplasm (PP), and clarified medium (CF) from each strain were resolved by SDS-PAGE, transferred onto nitrocellulose membranes, and incubated with MAb 1B5. The medium fraction was concentrated 10-fold before loading on SDS-PAGE.

Cargo proteins of the T9SS are anchored in the outer layer of the OM via covalently attached A-LPS. This type of LPS accumulates in the periplasm in the Δ*porV* mutant (33). To determine whether this is a general feature of secretion mutants that ablate translocation, we used the monoclonal antibody (MAb) 1B5, which exclusively recognizes a phosphorylated branched mannan (Manα1-2Manα1-phosphate) of A-LPS (10), to detect A-LPS in washed cells, isolated periplasm, and clarified growth medium from Δ*porZ* and Δ*porT* mutants, as well as their parental wild-type W83 strain. Δ*porV* fractions were used as positive controls, and strain HG66, which lacks A-LPS but produces intact O-LPS (34), was used as the negative control. Western-blot analysis revealed that all strains except HG66 had A-LPS in the whole-cell extract (Fig. 2, WC). In stark contrast, A-LPS was detected at high levels only in the periplasm of the secretion mutants but not in that of the wild-type W83 strain (Fig. 2, PP). Conversely, A-LPS was detected exclusively in the medium of W83 (Fig. 2, CF). Notably, A-LPS moieties in the periplasm of the secretion mutants had lower molecular masses than those from the whole-cell extract of the wild type (Fig. 2, WC and PP). This observation suggests that protein-depleted A-LPS of 35 to 40 kDa accumulated exclusively in the periplasm of the mutant strains. The lack of a protein component in this fraction was confirmed by treatment with proteinase K, which did not affect SDS-PAGE mobility of A-LPS (see Fig. S1A in the supplemental material). Conversely, when the whole-cell fraction of W83 was treated overnight with proteinase K, the high-molecular-weight band disappeared, confirming that at least some A-LPS in W83 is conjugated to proteins (see Fig. S1B). Finally, as expected, we observed no MAb 1B5-reactive material in any fraction derived from strain HG66 (Fig. 2, WC, PP, CF). Together, these data support the idea that secretion mutants retain fairly homogenous, protein-unconjugated A-LPS in the periplasm, whereas in the wild-type strain W83 the proteins are attached to the OM via A-LPS molecules.

10.1128/mBio.02262-20.1 FIG S1 A-LPS is conjugated to cargo proteins only in the wild-type strain W83. (A and B) Proteinase K treatment of periplasmic fraction (A) and washed cells (B) of P. gingivalis mutants and wild-type strain W83. The fractions were incubated at 60°C for 1 h, treated overnight with proteinase K, and then subjected to western-blot with MAb 1B5. PK, proteinase K. Download FIG S1, TIF file, 0.7 MB. Copyright © 2021 Madej et al. 2021 Madej et al. https://creativecommons.org/licenses/by/4.0/ This content is distributed under the terms of the Creative Commons Attribution 4.0 International license.

10.1128/mBio.02262-20.2 FIG S2A Sequence alignments for PorZ from P. gingivalis W83 and T. forsythia. The secondary structure assignment, based on the crystal structure of PorZ from P. gingivalis, is indicated. The image was produced by ESPript 3.01 (49). Sequence alignment was performed using Clustal Omega (50). Download FIG S2A, TIF file, 2.1 MB. Copyright © 2021 Madej et al. 2021 Madej et al. https://creativecommons.org/licenses/by/4.0/ This content is distributed under the terms of the Creative Commons Attribution 4.0 International license.

10.1128/mBio.02262-20.3 FIG S2B1 Sequence alignments for PorU from P. gingivalis W83 (residues 1 to 895) and T. forsythia (residues 1 to 926). The image was produced by ESPript 3.01 (49). Sequence alignment was performed using Clustal Omega (50). Download FIG S2B1, TIF file, 1.8 MB. Copyright © 2021 Madej et al. 2021 Madej et al. https://creativecommons.org/licenses/by/4.0/ This content is distributed under the terms of the Creative Commons Attribution 4.0 International license.

10.1128/mBio.02262-20.4 FIG S2B2 Sequence alignments for PorU from P. gingivalis W83 (residues 896 to 1123) and T. forsythia (residues 927 to 1153). The image was produced by ESPript 3.01 (49). Sequence alignment was performed using Clustal Omega (50). Download FIG S2B2, TIF file, 0.5 MB. Copyright © 2021 Madej et al. 2021 Madej et al. https://creativecommons.org/licenses/by/4.0/ This content is distributed under the terms of the Creative Commons Attribution 4.0 International license.

10.1128/mBio.02262-20.5 FIG S2C Specificity of PorZ from P. gingivalis. Fluorescently-labeled PorZ from *T. forsythia* was titrated with increasing concentrations of purified A-LPS from W83. The results are presented as means ± the SD from three experiments. Download FIG S2C, TIF file, 0.7 MB. Copyright © 2021 Madej et al. 2021 Madej et al. https://creativecommons.org/licenses/by/4.0/ This content is distributed under the terms of the Creative Commons Attribution 4.0 International license.

### The type IX secretion system does not transport the A-LPS to the outer membrane.

**FIG 3:**
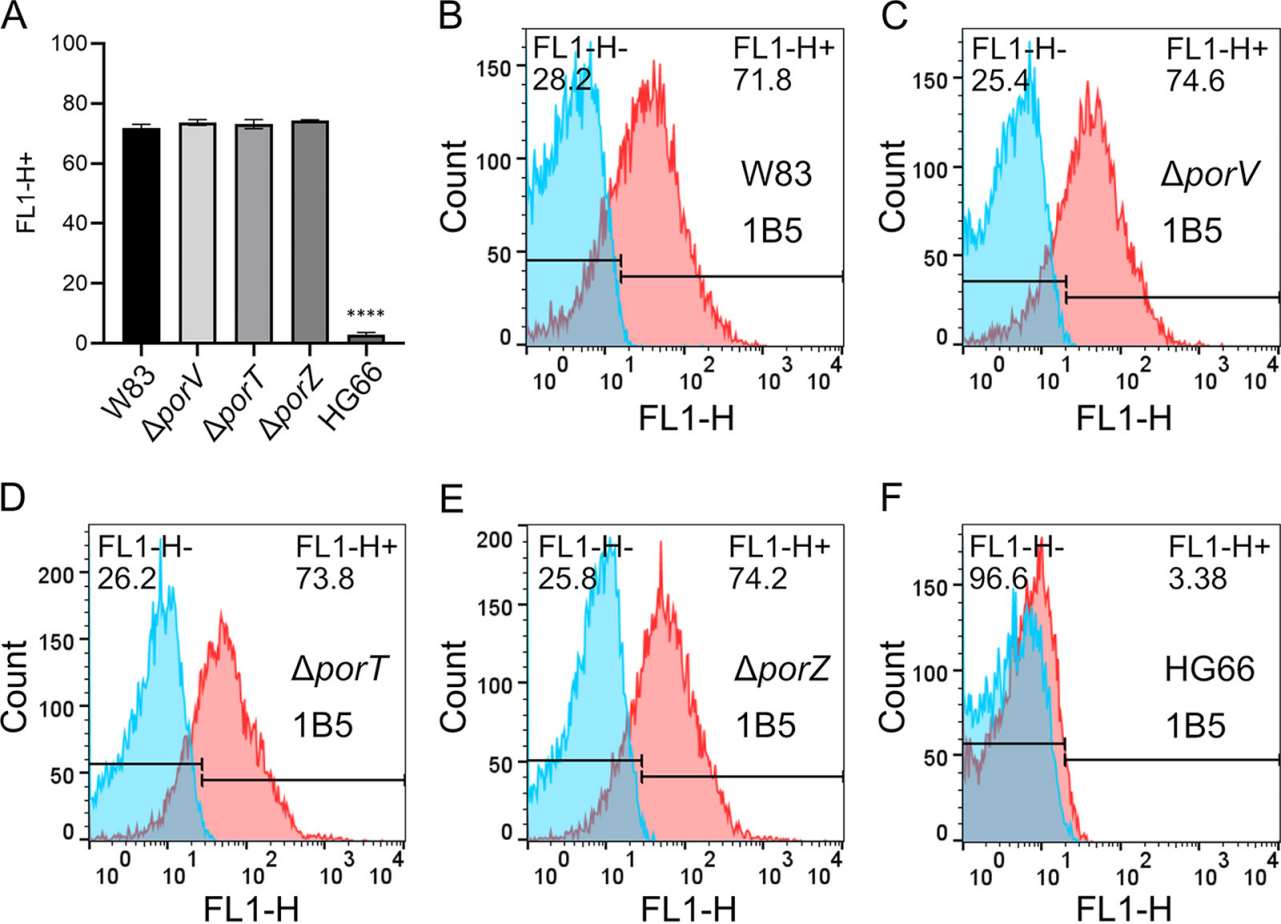
Exposure of A-LPS on the outer membrane of P. gingivalis. Flow cytometry using MAb 1B5 antibodies to determine surface exposure of A-LPS in Δ*porV*, Δ*porT*, Δ*porZ* mutants, and in W83 and HG66 wild-type strains. (A) A-LPS surface exposure as mean percentage of positive cells for the indicated strains, calculated based on the flow cytometry of three different cultures. Differences between the wild-type W83 parental strain and the secretion mutants were analyzed by one-way analysis of variance with Bonferroni’s correction. ****, *P* < 0.0001. (B to F) Representative histograms showing the percentage of MAb 1B5-labeled cells (shown in red) relative to the negative antibody isotype control (shown in blue).

Periplasmic accumulation of the A-LPS may affect the amount of A-LPS in the cell envelope. Therefore, to quantitatively compare the levels of A-LPS in the OM of the secretion mutants and wild-type strain, we analyzed live bacterial cells by flow cytometry using MAb 1B5 (Fig. 3). The specificity of the analysis was verified by the absence of staining in strain HG66 (Fig. 3F). In contrast, cells of the W83, Δ*porV*, and Δ*porT* strains reacted strongly with the antibody, with no clear difference in the abundance of A-LPS among the three (Fig. 3B to E). This result indicates that inactivation of genes essential for T9SS function has no significant impact on the level of A-LPS in the OM, implying that the A-LPS is exported in a T9SS-independent manner, possibly by some variant of the MsbA/Lpt system (35).

### PorZ interacts with free periplasmic A-LPS from Δ*porZ*, Δ*porV*, and Δ*porT* mutants.

**FIG 4:**
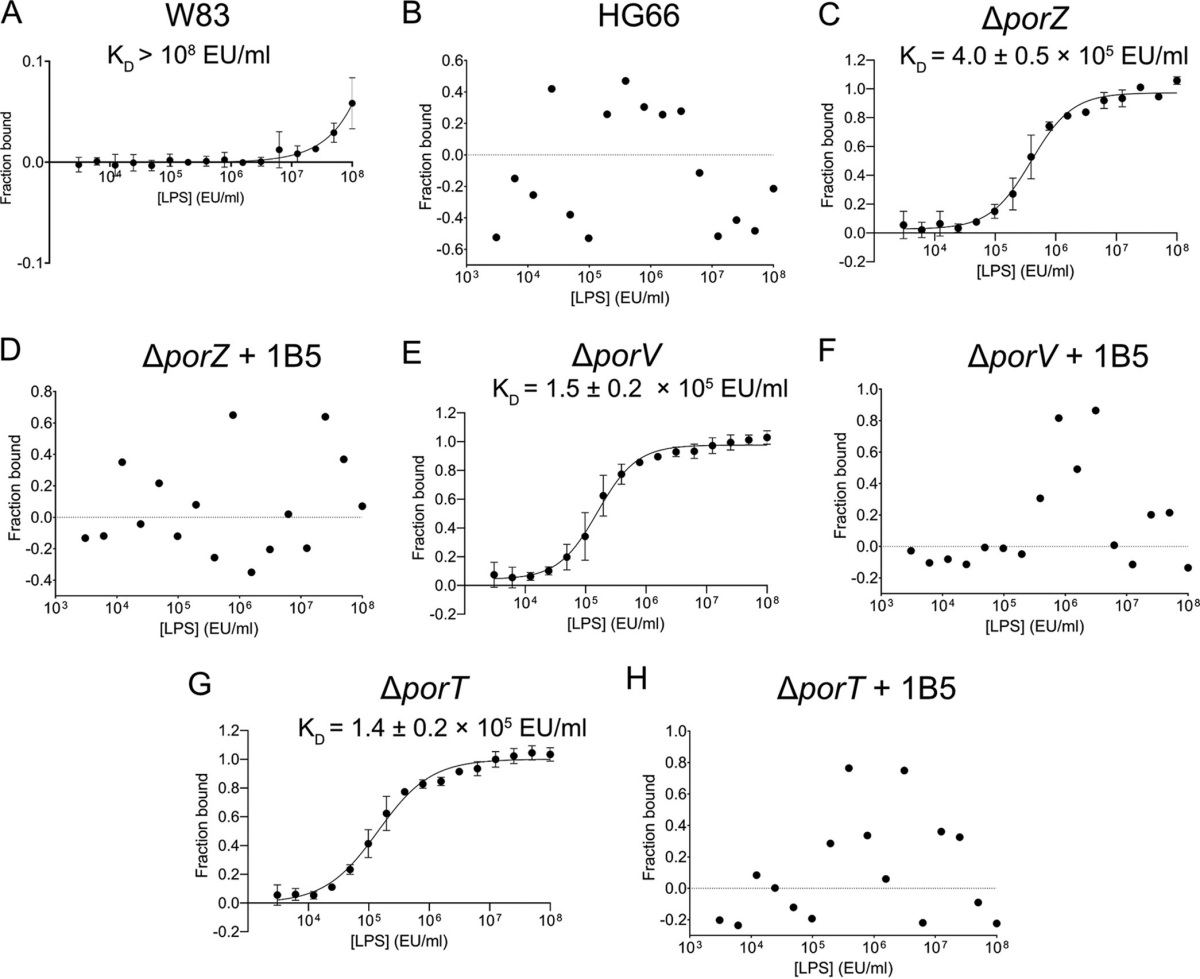
A-LPS in the periplasm of T9SS secretion mutants binds PorZ, and binding is blocked by MAb 1B5. Fluorescently-labeled PorZ was titrated with serial dilutions of the periplasmic fraction of W83 (A), HG66 (B), Δ*porZ* (C and D), Δ*porV* (E and F), and Δ*porT* (G, H) strains treated overnight with proteinase K, standardized to the same concentration of LPS based on an LAL assay, and then subjected to microscale thermophoresis analysis. To confirm the specificity of binding, periplasmic fractions were preincubated with MAb 1B5 (D, F, and H). Binding data were curve-fitted and used to determine *K_d_* values. The results are presented as the means ± the SD of three experiments.

We recently solved the crystal structure of full-length PorZ (32). The structure revealed that the protein consists of three domains: two seven-stranded β-propeller domains and a C-terminal seven-stranded β-sandwich domain. The latter resembles the canonical CTDs of other T9SS-secreted proteins, but in contrast to T9SS cargos, it is not cleaved by PorU. The β-propeller domains are often engaged in protein-protein and protein-ligand interactions (36). Furthermore, the overall structure of PorZ is similar to that of the periplasmic portion of histidine kinase BT4663, which is part of a two-component signal transduction system for the detection and degradation of complex carbohydrates in Bacteroides thetaiotaomicron, an abundant colonizer of the human gut (32, 37). In particular, both proteins comprise two N-terminal 7-fold β-propeller domains and a C-terminal all-β domain, dubbed the “Y_Y_Y domain” in BT4663. Based on these structural similarities, we hypothesized that PorZ may also have a glycan-binding function. Specifically, by binding A-LPS, it might be engaged in posttranslational modifications of T9SS cargos, resulting in OM anchorage. To test this hypothesis, we investigated whether PorZ could bind A-LPS in the periplasm of the Δ*porZ*, Δ*porV*, and Δ*porT* mutants. To this end, we treated periplasmic fractions overnight with proteinase K to remove proteins, concentrated them by ultrafiltration, and adjusted them to equivalent LPS content based on the *Limulus* amebocyte lysate (LAL) assay. We then quantitatively analyzed PorZ-LPS interaction by microscale thermophoresis. The experiments were performed with fluorescently-labeled PorZ and serial dilutions of each periplasmic fraction (Fig. 4). As expected, we observed no interaction with components of the HG66 periplasm (Fig. 4B) and only weak interaction with that of W83 (*K_d_* > 10^8^ endotoxin units [EU]/ml) (Fig. 4A). This is consistent with the absence of A-LPS in the cellular compartment of this strain, as determined by western-blot analysis (Fig. 2B, PP). By contrast, PorZ strongly bound A-LPS from the periplasm of Δ*porZ*, Δ*porV*, and Δ*porT* mutants with *K_d_* values of (4.0 ± 0.5) × 10^5^ EU/ml, (1.5 ± 0.2) × 10^5^ EU/ml, and (1.4 ± 0.2) × 10^5^ EU/ml, respectively. (Fig. 4C, E, and G). However, preincubation of the periplasm with MAb 1B5 completely abolished these interactions (Fig. 4D, F, and H). Given that MAb 1B5 specifically recognizes phosphorylated branched mannan (10), which is a mosaic component of A-PS within A-LPS, these results strongly argue that this unique saccharide moiety is the recognition motif of PorZ.

### PorZ interacts with purified A-LPS.

**FIG 5:**
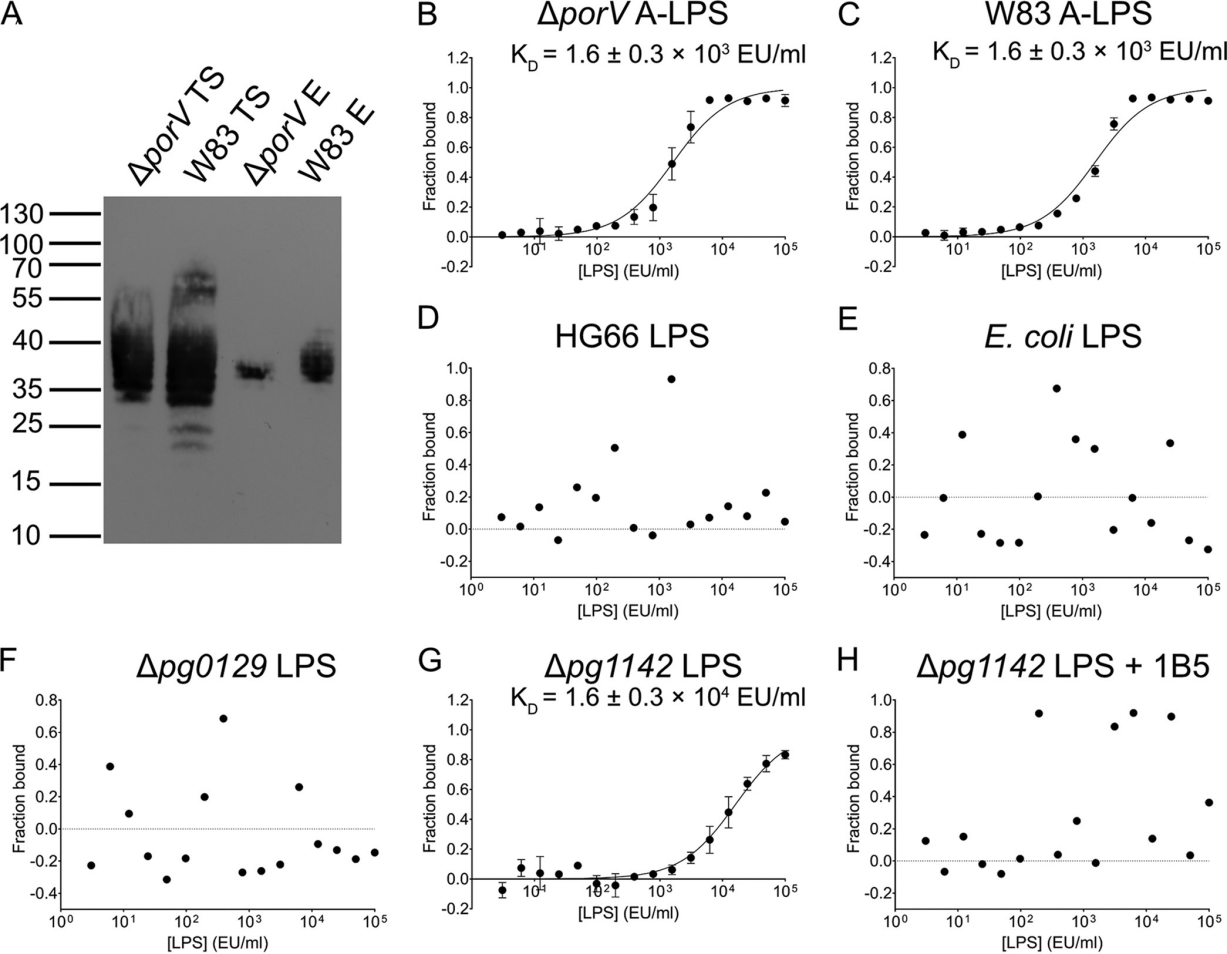
PorZ binds a subset of P. gingivalis W83-derived A-LPS, which is dependent on the presence of branched phosphomannan units in the A-polysaccharide. (A) GST-tagged PorZ immobilized on glutathione-Sepharose resin was incubated with purified A-LPS from Δ*porV* and W83 strains (loaded A-LPS), and the resin was extensively washed. PorZ, together with interacting A-LPS, was released by cleavage with PreScission protease and analyzed for the presence of A-LPS by western-blot using MAb 1B5 (PorZ-bound A-LPS). TS, total A-LPS sample from Δ*porV* used for incubation with GST-tagged PorZ; E, eluted fraction, i.e. A-LPS bound to PorZ eluted from the glutathione-Sepharose resin. (B) Microscale thermophoresis analysis of fluorescently-labeled PorZ incubated with serial dilutions of A-LPS purified from Δ*porV* (B) and W83 (C) strains or total LPS extracted from HG66 (D), E. coli (E), Δ*pg0129* (F), and Δ*pg1142* strains in the absence (G) or presence (H) of MAb 1B5. Binding data were curve-fitted and used to determine *K_d_* values. The results are presented as the means ± the SD of three experiments.

To verify that PorZ indeed binds A-LPS, we performed pulldown assays with PorZ in the presence of A-LPS extracted and purified from whole cells of the Δ*porV* mutant and the parental wild-type strain. To this end, we first incubated A-LPS with immobilized PorZ in an affinity chromatography column, followed by extensive washing and elution of PorZ, and then subjected the eluted fraction to western-blot analysis with MAb 1B5. Although A-LPS from whole Δ*porV* and wild-type cells had a wide range of sizes (35 to 55 kDa and 20 to 70 kDa, respectively), purified A-LPS migrated more homogeneously (35 to 40 kDa) (Fig. 5A), reminiscent of A-LPS species from the periplasmic fractions of the secretion mutants (Fig. 2, PP). Notably in this regard, no MAb 1B5-immunoreactive material was eluted from a column that was not charged with PorZ (data not shown).

The interaction between PorZ and purified A-LPS was further examined by microscale thermophoresis (Fig. 5B to H), as in the aforementioned experiments with periplasm-derived A-LPS. Fluorescently-labeled PorZ was titrated with serial dilutions of A-LPS purified from W83 and Δ*porV*. In the case of strain HG66 and the Δ*pg0129* and Δ*pg1142* mutants, total LPS (A-LPS and O-LPS) was extracted. The latter two mutants are deficient in α-1,3-mannosyltransferase and O-antigen polymerase activity, respectively (38). The Δ*pg0129* mutant synthesizes a deep-R-type LPS with a truncated core region and does not immunoreact with MAb 1B5 (39). In contrast, the Δ*pg1142* mutant does immunoreact with MAb 1B5 in western-blots because it makes SR-type LPS (core plus one repeated unit), containing both an SR-type O-LPS and SR-type A-LPS (39). A-LPS extracted from Δ*porV* and W83 strains interacted with PorZ with similar affinities [*K_d_* = (1.6 ± 0.3) × 10^3^ EU/ml], apparently because the concentrations of A-LPS in the two extracts were similar (Fig. 5B and C). Notably, the difference in *K_d_* between PorZ-binding periplasm (Fig. 4E) and the whole cell-derived A-LPS from the *porV* mutant strain (Fig. 5B) was the result of the normalization of the LPS content according to the LAL assay; the periplasm contains a much higher titer of O-LPS than purified A-LPS. In contrast, LPS from HG66 and Escherichia coli did not bind to PorZ (Fig. 5D and E), further corroborating the finding that regular O-LPS does not interact with PorZ. Remarkably, PorZ-bound A-LPS was produced by the Δ*pg1142* strain but not by the Δ*pg0129* strain (Fig. 5F and G). Because the difference between A-LPS molecules produced by the Δ*pg1142* and Δ*pg0129* strains is a single repeat of the phosphorylated branched mannan in SR-type A-LPS produced by the former but absent in the deep-R-type LPS synthesized by the latter, the phosphorylated branched mannan must be the structure involved in the relatively low-affinity interaction with PorZ [*K_d_* = (1.6 ± 0.3) × 10^4^ EU/ml]. In keeping with this contention, deep-R-type A-LPS produced by the Δ*pg0129* strain did not bind PorZ (Fig. 5F). Moreover, the interaction between Δ*pg1142* strain-derived A-LPS and PorZ was completely abolished in the presence of MAb 1B5 (Fig. 5H). Together, these results unambiguously argue that PorZ specifically recognizes the phosphorylated branched mannan moiety in A-LPS.

### PorZ preferentially interacts with PorU in the presence of A-LPS.

**FIG 6:**
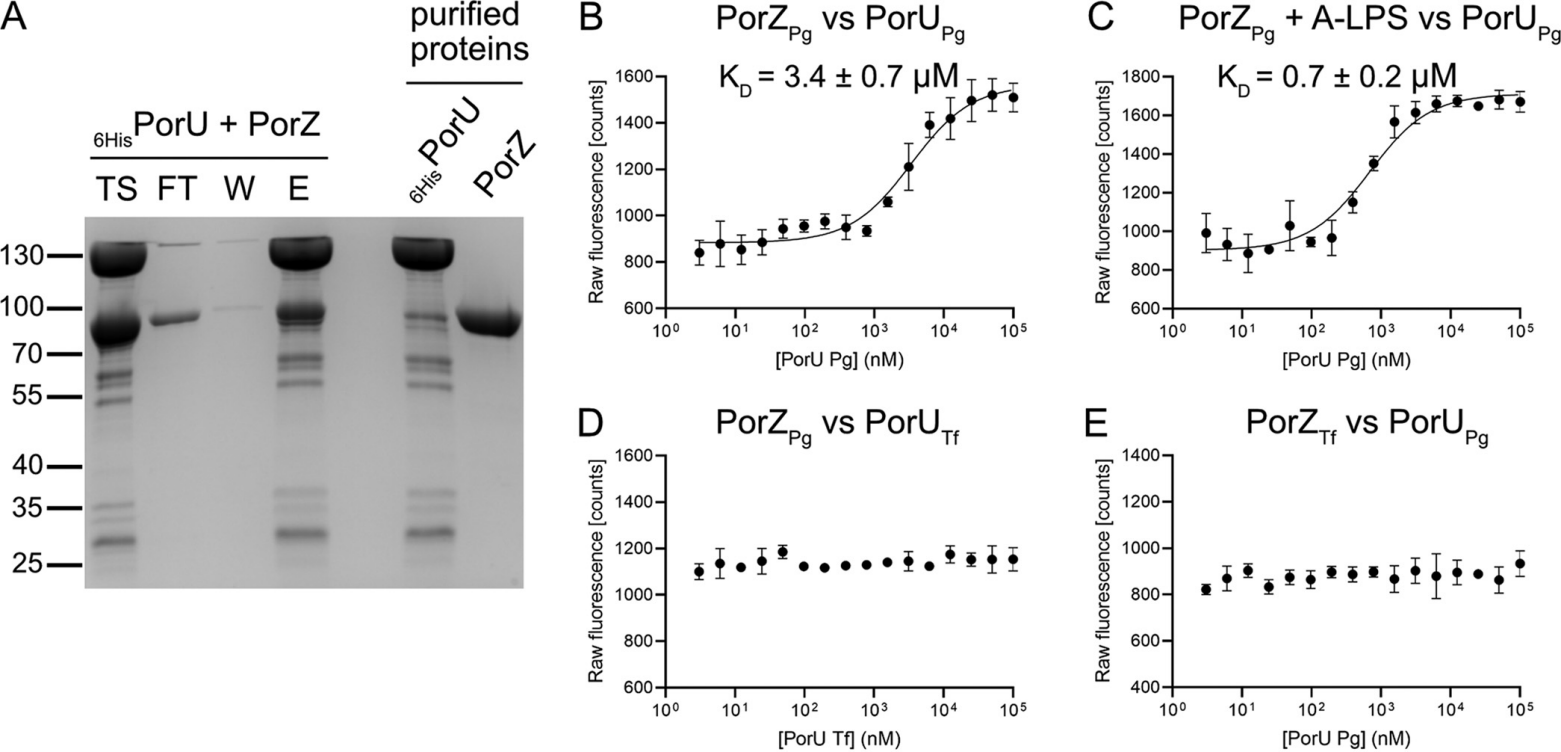
PorZ binds PorU sortase with an affinity that is enhanced by A-LPS. (A) In a pull-out assay, purified recombinant proteins 6His-PorU and PorZ (right two lanes) were mixed and incubated with cobalt-based magnetic beads. The beads were washed, and the bound protein was eluted. The resulting fractions were analyzed by SDS-PAGE and Coomassie blue staining. TS, sample before loading on beads; FT, flowthrough; W, bead wash; E, eluted proteins. (B to D) Fluorescently-labeled PorZ from P. gingivalis (PorZ_Pg_) was titrated with increasing concentrations of PorU from P. gingivalis (PorU_Pg_) without A-LPS (B) in the presence of 1 × 10^5^ EU/ml A-LPS from W83 (C) or titrated with increasing concentrations of PorU from T. forsythia (PorU_Tf_). In a reciprocal experiment, fluorescently-labeled PorZ from *T. forsythia* (PorZ_Tf_) was titrated with increasing concentrations of PorU from P. gingivalis (PorU_Pg_). (E) Binding data were curve-fitted and used to determine *K_d_* values. The results are presented as the means ± the SD of three experiments.

Recently, we showed that surface exposure of PorU is dependent on the presence of PorZ (32). To determine whether this dependence results from a direct interaction between both molecules, we expressed and purified both proteins and performed copurification analysis. Most PorZ bound immobilized His-tagged PorU, indicating that the two proteins interact (Fig. 6A). To confirm this interaction, we performed microscale fluorescence analysis. In these experiments, fluorescently-labeled PorZ was titrated with serial dilutions of PorU in the absence (Fig. 6B) or presence (Fig. 6C) of purified A-LPS from W83. In the presence of A-LPS, the affinity of PorZ for PorU increased 5-fold, as indicated by the difference in the respective dissociation constants (*K_d_* = 0.7 ± 0.2 μM or 3.4 ± 0.7 μM in the presence or absence of A-LPS, respectively). The stronger binding of A-LPS-loaded PorZ to PorU may play a crucial role in the attachment of A-LPS to the C-terminal residue of T9SS cargos during cleavage of the CTD by PorU.

### PorU-PorZ interactions are species specific.

T9SS is conserved in many Bacteroidetes species, including Tannerella forsythia, which is strongly implicated in the pathogenesis of chronic periodontitis (40). Like P. gingivalis, *T. forsythia* secretes many virulence factors via a T9SS and anchors them in the OM (41). To compare the function of PorU and PorZ in these two periodontal pathogens, we investigated reciprocal interactions between these two proteins in homogenous and heterologous systems. Interestingly, neither *T. forsythia* protein cross-reacted with its P. gingivalis ortholog (Fig. 6D and E). Although this lack of cross-reactivity can be attributed to the relatively low level of conservation of the primary structures (see Fig. S2A, S2B1, S2B2, and S2C), it rather results from distinct functions of the two systems since *T. forsythia* PorZ does not bind A-LPS from P. gingivalis (see Fig. S2C). This specialization is likely related to the lack of A-LPS or generally a polysaccharide containing a phosphorylated branched mannan in *T. forsythia*, as indicated by the lack of MAb 1B5 binding to *T. forsythia* extracts (42). It is tempting to speculate that in this bacterium PorZ assists PorU in the attachment of T9SS cargo proteins to alternative OM lipids different from A-LPS of P. gingivalis.

## DISCUSSION

P. gingivalis contains two different lipopolysaccharides (LPS) in its OM: O-LPS and A-LPS (9). The main difference between them is the chemical composition of the O-antigen. The tetrasaccharide repeat unit in O-LPS consists of a conventional polysaccharide (PS), which is distinct from the anionic polysaccharide (APS) repeat unit in A-LPS (Fig. 1). The APS unit contains a phosphorylated branched mannan specifically recognized by MAb 1B5 (10). This serves as a convenient tool for visualizing A-LPS alone or conjugated to T9SS cargo proteins by a variety of immunological methods (33). Using this approach, we showed that P. gingivalis mutants with dysfunctional T9SS accumulated A-LPS in a protein-unconjugated form in the periplasm, regardless of which component of the T9SS machinery was inactivated by mutagenesis (Fig. 2, PP).

A-LPS accumulated in the periplasm of Δ*porZ*, Δ*porV*, and Δ*porT* strains and interacted with PorZ. The dissociation constant (*K_d_*), determined by microscale thermophoresis, differed from sample to sample, suggesting variations in the A-LPS concentration (Δ*porT* > Δ*porV* > Δ*porZ*) in the periplasm of these secretion mutants (Fig. 4). The *K_d_* calculation was based on the concentration of total LPS estimated using the LAL assay, with samples adjusted to the same titer of LPS regardless of the A-LPS/O-LPS ratio. Consequently, the values obtained are only semiquantitative. Nevertheless, the general usefulness of the assay was validated by the fact that PorZ did not interact with the periplasm derived from strain HG66, which produces intact O-LPS but lacks A-LPS (34), and interacted very weakly with periplasm derived from wild-type P. gingivalis. These observations correlate perfectly with the absence of immunoreactive A-LPS in these fractions (Fig. 2). In the latter case, the interactions detected by MST were probably due to periplasmic contamination with OM-derived A-LPS acquired during subcellular fractionation.

A previous study reported that PorV has lipid A deacylase activity, and the Δ*porV* mutant produces only penta-acylated forms of monophosphorylated lipid; in contrast, wild-type P. gingivalis and the Δ*porT* mutant both produce a tetra-acylated form of monophosphorylated lipid A in addition to the penta-acylated form (33). The comparable *K_d_* values of PorZ interactions with A-LPS in periplasm derived from these strains clearly suggests that even if PorV has deacylase activity, which was questioned by a subsequent study (43), the acylation profile of lipid A has no influence on this binding. Also, differences in lipid A phosphorylation, which appear to be influenced by PorV (43), did not have any effect on the interaction between A-LPS and PorZ, since the *K_d_* values were comparable for all tested secretion mutants. Hence, A-LPS binding to PorV may be independent of the structure of lipid A. Of note, the structure of PorV in complex with the Sov translocon from Flavobacterium johnsoniae was solved recently (28). Although it is controversial whether PorV possesses lipid A modifying activity in addition to its role as a shuttle protein in the T9SS system, it cannot be ruled out that PorV is able to exert an indirect effect on the structure of lipid A of A-LPS attached to T9SS cargo proteins through the recruitment/activation of lipid A phosphorylases, as suggested previously (43).

Together, these observations indicate that A-LPS, regardless of its lipid A acylation and/or phosphorylation status, binds specifically to PorZ. The presence of A-LPS in the periplasm of secretion mutants was in stark contrast to the wild-type strain, which almost completely lacked A-LPS in the periplasm. Remarkably, periplasmic accumulation of A-LPS in secretion mutants did not affect its level in the OM (Fig. 3). This is intriguing in the context of LPS biosynthesis and transport from the cytoplasm across the cellular envelope to the bacterial surface.

The lipid A-core oligosaccharide complex is synthesized on the cytoplasmic face of the IM and flipped to the periplasmic side of the IM (44). Here, O-antigen, which is independently synthesized in the cytoplasm and translocated to the periplasmic face of the IM as undecaprenol phosphate-linked O-antigen (in P. gingivalis A-PS or O-PS), is ligated to lipid A-core oligosaccharide by the IM-associated WaaL ligase. Then, the mature LPS (in P. gingivalis O-LPS and A-LPS) is transported to the cell surface by the Lpt (LPS transport) pathway, which involves several essential proteins that bridge IM and OM in the periplasm (45). Notably in this regard, all Lpt components are conserved in P. gingivalis. The transport occurs in a continuous manner (the PEZ model), without any LPS leaking into the periplasm (35). Therefore, the presence of considerable amounts of A-LPS in the periplasm of P. gingivalis T9SS mutants is surprising because it suggests the existence of a selective shunt of A-LPS into the periplasm from the continuous flow of the molecules along the Lpt periplasmic bridge. The distinct size of A-LPS in the periplasm (Fig. 2, PP) and its interaction with PorZ (Fig. 5A), in contrast to A-LPS extracted from whole cells (Fig. 2, WC), argues that two distinct pools of A-LPS exist. It is tempting to speculate that this is related to additional modification of the A-LPS that is ultimately attached to secreted proteins via a proposed 648-Da linker (46). The linker may provide an amino group for nucleophilic attack on the thioester intermediate in the PorU-catalyzed proteolysis of a peptide bond that leads to removal of the CTD and attachment of A-LPS to the C terminus of a truncated T9SS cargo protein (46). Because PorU is localized on the bacterial surface, this transpeptidation reaction must occur after A-LPS, with its linker, translocates to the OM, where it encounters T9SS cargo proteins and PorU. In T9SS mutants, translocation is apparently dysfunctional, leading to accumulation of A-LPS in the periplasm.

An elegant recent study by the Reynolds group showed that the surface-localized (PorU and PorZ) and integral OM β-barrel proteins (PorV and PorQ) form a 440-kDa attachment complex (29). In their model, PorV serves to shuttle T9SS cargo proteins from the OM translocon to the attachment complex. Once transferred to the attachment complex the PorU sortase cleaves the CTD and attaches A-LPS, provided by PorZ, to T9SS cargo proteins, thereby facilitating anchorage to the OM. Our results experimentally validate this model by showing that A-LPS does indeed interact with PorZ. The signature pattern recognized by PorZ was unambiguously mapped to a phosphorylated branched mannan (Manα1-2Manα1-phosphate) in APS (A-PS) (Fig. 4 and 5). A single repeat unit of A-LPS is sufficient to interact with PorZ, but with decreased affinity (Fig. 5G) that is not sufficient to enable attachment of A-LPS to secreted proteins. The P. gingivalis Δ*pg1142* mutant strain, which produces severely truncated A-PS carrying a single repeat of the phosphorylated branched mannan, is unable to retain gingipains on the cell surface and instead releases them in unmodified, soluble forms into the medium (38).

We also showed for the first time that PorZ directly interacts with PorU, and that this interaction was significantly strengthened in the presence of A-LPS (Fig. 6B and C). This change in affinity may be essential for A-LPS transfer from PorZ to T9SS client proteins mediated by the sortase activity of PorU. Once CTD is cleaved and released into the growth medium (30) and A-LPS is attached to a cargo protein, both PorV and PorZ are recycled for another round of coordinated transport and attachment.

Taken together, our findings not only complement the previously proposed model of molecular events occurring on the P. gingivalis surface during protein secretion through T9SS (29) but also add significant mechanistic insights. First, our data argue that A-LPS designated for attachment to T9SS cargo proteins is probably transported to the cell surface via a periplasmic/OM shunt independent of the Lpt pathway of O- and A-LPS insertion into the outer layer of the OM. Second, PorZ functions as the A-LPS shuttle protein, specifically recognizing the phosphorylated branched mannan in the repeat units of the A-PS. Third, the enhanced affinity of A-LPS loaded-PorZ for PorU facilitates presentation of A-LPS to the sortase. Subsequent cleavage of the CTD from the T9SS cargo protein by the sortase simultaneously catalyzes a transpeptidation reaction that attaches the C-terminal carbonyl group of the cargo protein via an isopeptide bond (46) to A-LPS positioned at the surface of the bacterium.

## MATERIALS AND METHODS

### Bacterial strains and general growth conditions.

P. gingivalis strains (wild-types W83 and HG66 and mutants; listed in Table S1 in the supplemental material) were grown in enriched tryptic soy broth (eTSB; 30 g Trypticase soy broth and 5 g yeast extract per liter at pH 7.5; further supplemented with 5 mg hemin, 0.5 g l-cysteine, and 2 mg menadione) or on eTSB blood agar (eTSB medium containing 1.5% [wt/vol] agar; further supplemented with 4% defibrinated sheep blood) at 37°C in an anaerobic chamber (Don Whitley Scientific, UK) with an atmosphere of 90% nitrogen, 5% carbon dioxide, and 5% hydrogen. Escherichia coli strains (listed in Table S2), used for all plasmid manipulations, were grown in Luria-Bertani (LB) medium and on 1.5% LB agar plates. For antibiotic selection in E. coli, ampicillin was used at 100 μg/ml. P. gingivalis mutants were grown in the presence of erythromycin at 5 μg/ml and/or tetracycline at 1 μg/ml.

10.1128/mBio.02262-20.6 TABLE S1 P. gingivalis strains used in this study. Download Table S1, DOCX file, 0.02 MB. Copyright © 2021 Madej et al. 2021 Madej et al. https://creativecommons.org/licenses/by/4.0/ This content is distributed under the terms of the Creative Commons Attribution 4.0 International license.

10.1128/mBio.02262-20.7 TABLE S2 E. coli strains and plasmids used in this study. Download Table S2, DOCX file, 0.02 MB. Copyright © 2021 Madej et al. 2021 Madej et al. https://creativecommons.org/licenses/by/4.0/ This content is distributed under the terms of the Creative Commons Attribution 4.0 International license.

### Generation of Δ*porZ*, Δ*porT*, and Δ*porV* deletion mutants of *P. gingivalis*.

The P. gingivalis Δ*porZ* and Δ*porT* deletion mutants were generated by homologous recombination, as previously described (32, 47). The Δ*porV* mutant was created using the same strategy. In brief, two 1-kb segments of DNA flanking the *porV* gene in P. gingivalis W83 (TIGR accession number PG0027) were amplified by PCR using Accuprime Pfx DNA polymerase (Invitrogen) and the primer pairs PG23FrBXbaIF/PG23FrBHind3R and PG23FrASacIF/PG23FrASmaR (the sequences are listed in Table S3) and ligated into the multiple cloning site of the pUC19 plasmid at XbaI/HindIII and SacI/SmaI sites, respectively. An erythromycin-resistance cassette, *ermF-ermAM*, was amplified by PCR from pVA2198 (48) using the primer pair ermFAMSmaIF/ermFAMXbaIR and ligated between the flanking DNA segments at SmaI and XbaI sites to create the final plasmid, p23AeB-B. Then, 1 μg of p23AeB-B was electroporated into electrocompetent P. gingivalis cells to allow for homologous recombination of the construct into the genome to result in deletion of *porV* and acquisition of the *ermF-ermAM* cassette. Erythromycin-resistant P. gingivalis colonies were subsequently confirmed for deletion of *porV* via PCR amplification and sequencing of the pertinent region of the genome. *pg0129* (α-1,3-mannosyltransferase) and *pg1142* (Wzy, O-antigen polymerase) deletion mutants were constructed and characterized by Rangarajan et al. (38). The deletion of *pg1142* leads to a core-plus-one repeating unit structure for both LPS types, whereas the inactivation of *pg0129* leads to the absence of A-PS and O-PS moieties.

10.1128/mBio.02262-20.8 TABLE S3 Primers used in this study. Download Table S3, DOCX file, 0.02 MB. Copyright © 2021 Madej et al. 2021 Madej et al. https://creativecommons.org/licenses/by/4.0/ This content is distributed under the terms of the Creative Commons Attribution 4.0 International license.

### Subcellular fractionation of *P. gingivalis* strains.

Stationary-phase cultures of wild-type P. gingivalis and mutants were adjusted to an optical density at 600 nm (OD_600_) of 1.0, and cells were collected by centrifugation (8,000 × *g*, 15 min). The cell pellet was then washed and resuspended in phosphate-buffered saline (PBS). This fraction is referred to as washed cells (WC). The collected cell-free culture medium was ultracentrifuged (100,000 × *g*, 1 h) to remove vesicles, and the supernatant was concentrated 10-fold by ultrafiltration using 3-kDa cutoff centricones (EMD, Millipore, Billerica, MA); this fraction was designated as the clarified medium (CF). The WC fraction, obtained as described above, was suspended in buffer containing 0.25 M sucrose and 30 mM Tris-HCl at pH 7.6. After a 10-min incubation period, the cells were pelleted (12,500 × *g*, 15 min) and rapidly resuspended in 2.5 ml of cold distilled water to disrupt the OM. After an additional 10-min incubation period, spheroplasts were pelleted by centrifugation (12,500 × *g*, 15 min). The supernatant was collected and designated as the periplasmic (PP) fraction. All fractions were supplemented with peptidase inhibitors (5 mM tosyl-l-lysyl-chloromethyl ketone [TLCK], 1 mM 2,2′-dithiodipyridine [DTDP], 1× EDTA-free protein inhibitor cocktail; all from Roche) before storage at –20°C.

### Western-blot analysis.

Samples of the washed cells, PP fraction, and medium fraction were resolved by SDS-PAGE and transferred to a nitrocellulose membrane. To visualize the total protein transferred, Ponceau S staining was used. After washing out Ponceau S with H_2_O, the membranes were blocked with 5% nonfat skim milk in TBST (0.1% [vol/vol] Tween 20 in 20 mM Tris [pH 7.5], 500 mM sodium chloride) overnight at 20°C, incubated with primary antibody 1B5 (1 μg/ml) for 2 h at room temperature, followed by incubation with goat anti-mouse horseradish peroxidase-conjugated IgG (1/20,000 dilution) in blocking solution for 1 h at room temperature. Development was carried out with an ECL western-blotting substrate kit according to the manufacturer’s instructions (Pierce, UK).

### Digestion of *P. gingivalis* proteins in whole-cell extract and the periplasmic fraction by proteinase K.

The aliquots of the PP fraction and whole-cell extract were heated at 60°C for 1 h. Then, the samples were cooled on ice, and proteinase K (Promega) was added to a final concentration of 100 μg/ml. The samples were incubated at 37°C overnight and then subjected to western-blotting with MAb 1B5.

### Isolation of anionic lipopolysaccharide and total lipopolysaccharide.

Isolation of A-LPS from the Δ*porV* mutant and W83 strain was performed according to a previously described procedure (10). For affinity chromatography, ion-exchange chromatography, and size exclusion chromatography, HiTrap ConA 4B, HiPrep DEAE FF 16/10, and Superdex200 Increase 10/300 GL columns, respectively, were used. All columns were purchased from GE Healthcare Life Sciences. Isolation of total LPS from HG66, Δ*pg0129*, and Δ*pg1142* strains was carried out using an LPS extraction kit (Intron Biotechnology). E. coli LPS was purchased from Sigma. The concentration of LPS was determined using a *Limulus* amebocyte lysate kit (Lonza) and shown in endotoxin units (EU/ml) calculated based on a standard curve.

### Flow cytometry analysis.

To reduce the low background of MAb 1B5 on HG66 cells, the antibodies at a concentration of 200 μg/ml were first incubated with PBS-washed HG66 cells (OD_600_ = 0.2) for 1 h at room temperature. The cells were then centrifuged (6,000 × *g*, 15 min), and the supernatant, devoid of nonspecific antibodies, was filtered through a 0.2-μm syringe filter. The concentration of antibodies was adjusted to 40 μg/ml and used for flow cytometry analysis. P. gingivalis strains were grown in eTSB until they reached the late exponential stationary growth phase (OD_600_ = 1.2 to 1.5). Bacterial cells were harvested by centrifugation, washed twice with PBS, and adjusted to an OD_600_ of 1.0 with staining buffer (PBS supplemented with 2.5% bovine serum albumin). Then, 100 μl of the cell suspension was transferred to a 96-well conical plate and incubated for 30 min with staining buffer. Cells were collected by centrifugation (500 × *g*, 5 min), and the pellet was resuspended in the staining buffer containing MAb 1B5 at a total protein concentration of 40 μg/ml, followed by incubation for 30 min. Thereafter, cells were centrifuged (500 × *g*, 5 min), and the newly obtained pellet was resuspended in the staining buffer containing goat anti-mouse antibody conjugated with fluorescein isothiocyanate (Abcam) at a 1:200 dilution and incubated for 30 min. The cells were washed twice with PBS after each incubation with antibodies. The whole staining procedure was performed on ice. After staining, one-color flow cytometry analyses were performed using a FACSCalibur apparatus (BD Biosciences) operating with CellQuest software (BD Biosciences). Graphs were prepared using the FLOWJO v.10.6.2 program (Ashland).

### Expression and purification of PorZ and PorU.

PorZ was expressed and purified as previously described (32). PorU was expressed and purified from E. coli. Briefly, a gene encoding PorU lacking the predicted signal peptide (M^1^-A^23^) was amplified by PCR, purified, and cloned into the pETDuet-1 expression vector. The expression plasmid was transformed into the E. coli strain BL21(DE3). Transformed cells were grown in Luria-Bertani medium with ampicillin (100 μg/ml) at 37°C until reaching an OD_600_ of approximately 0.2 to 0.3, and then protein expression was induced with 0.2 mM IPTG (isopropyl-β-d-thiogalactopyranoside) and allowed to proceed for 16 h at 20°C. The cells were lysed by sonication and centrifuged (40 min, 40,000 × *g*, 4°C). The supernatant was loaded on preequilibrated Nickel Sepharose 6 Fast Flow resin (GE Healthcare Life Sciences), the resin was washed, and the protein was eluted with 20 mM trisodium phosphate, 0.5 M sodium chloride, 250 mM imidazole, and 0.02% sodium azide (pH 7.4). PorU was further purified by size exclusion chromatography using a HiLoad 16/600 Superdex 200-pg column (GE Healthcare) equilibrated with 5 mM Tris-HCl, 50 mM sodium chloride, 0.02% sodium azide (pH 8.0), and an ÄKTA pure FPLC system (GE Healthcare). PorZ and PorU from *Tannerella forsythia* were expressed and purified in a similar manner. Protein concentrations were determined by measurement of absorbance at 280 nm using a NanoDrop device (Thermo Fisher Scientific) using a theoretical Abs 0.1% value (1.01) calculated by ProtParam (http://web.expasy.org/).

### Analysis of the interaction between A-LPS and PorZ by microscale thermophoresis.

Periplasmic fractions of W83, HG66, Δ*porZ*, Δ*porV*, and Δ*porT* strains were prepared as described earlier. The fractions were assayed for the LPS concentration using the LAL kit (Lonza) and adjusted to the same LPS content. The PP fractions were treated overnight with proteinase K and then ultrafiltrated with filter devices with a 3-kDa cutoff (Merck Millipore). Proteinase K was then thermally inactivated. A Monolith NT.115 instrument (NanoTemper Technologies GmbH, Munich, Germany) was used to analyze the binding interactions between the recombinant PorZ, and the LPS was purified from the periplasmic space. PorZ was fluorescently labeled on lysine residues with NT-647 NHS dye. The PP fraction with LPS concentrations ranging from 3 × 10^3^ to 1 × 10^8^ EU/ml was incubated for 5 min at 20°C with 50 nM PorZ. Experiments were performed in PBS at pH 7.4 at an ambient temperature of 25°C. The samples were loaded into Monolith NT.115 standard treated glass capillaries, and initial fluorescence measurements followed by thermophoresis measurements were carried out using 40% LED power and medium MST power. *K_d_* values were calculated using MO.Affinity Analysis software assuming 1:1 binding model and using the signal from default MST-on time. Experiments were performed in triplicate. Binding experiments with purified A-LPS from the Δ*porV* mutant and the W83 wild-type strain, commercially available LPS from E. coli, and total LPS isolated from P. gingivalis HG66, Δ*pg0129*, and Δ*pg1142* were performed analogically. The concentration of LPS used was in the range of 3 × 10^1^ to 1 × 10^5^ EU/ml.

### Analysis of the interaction between PorZ and PorU by microscale fluorescence.

A Monolith NT.115 was used to measure the binding interactions between PorU and PorZ or PorZ saturated with A-LPS from the W83 strain. PorU at a concentration of 3 nM to 100 μM was incubated for 5 min with 20 nM PorZ in PBS. Alternatively, A-LPS from W83 at a concentration of 1 × 10^5^ EU/ml was added to PorZ, which was then incubated with PorU. All measurements were performed at an ambient temperature of 25°C. The samples were loaded into the Monolith NT.115 premium treated glass capillaries, and initial fluorescence measurements were carried out using 60% LED power. A concentration-dependent increase in fluorescence of NT-647-labeled PorZ in the serial dilution of PorU was observed. Therefore, a “standard deviation (SD) test” was performed to discriminate between binding-specific fluorescence quenching and loss of fluorescence due to aggregation of PorZ upon addition of PorU. To this end, samples corresponding to low and high PorU concentrations (3, 6, and 12 nM and 25, 50, and 100 μM, respectively) were first centrifuged for 10 min at 16,000 × *g* to remove protein aggregates and then mixed with a 2× solution containing 4% SDS and 40 nM dithiothreitol and heated to 95°C for 5 min to denature PorZ. Subsequently, the fluorescence of these samples was measured using a Monolith NT.115 instrument. If the increase in fluorescence in the serial dilution is binding induced, the fluorescence intensities after denaturation should be identical, independent of the titrant concentration, which was the case for PorU-PorZ interactions regardless of the presence of A-LPS. *K_d_* values were calculated using the MO.Affinity Analysis software assuming 1:1 binding model. Experiments were performed in triplicate. Since the *K_d_* values for this interaction were determined based on raw fluorescence data, the method has been called “microscale fluorescence.”

### Pulldown assay.

GST-tagged PorZ was expressed and purified from E. coli as previously described (32) and then immobilized on glutathione-Sepharose resin (5 mg of protein per 5 ml of resin). Immobilized GST-PorZ was incubated with purified A-LPS from Δ*porV* and W83 strains for 2 h at 4°C, and the resin was washed extensively with PBS. PorZ, together with interacting A-LPS, was released by cleavage with PreScission protease (1 mg PreScission protease per 5 ml of resin) and analyzed for the presence of A-LPS by western-blot using MAb 1B5 as described earlier in Materials and Methods.

### Copurification assay.

The purified PorZ and hexahistidine-tagged PorU were mixed in equimolar concentration and incubated on ice for 10 min. The mixture was then applied to Co^2+^-nitrilotriacetic acid (Co^2+^-NTA) magnetic beads (Novex) in 100 mM sodium phosphate (pH 8.0), 600 mM sodium chloride, and 0.02% Tween 20 and then incubated at 25°C for 10 min. The beads were washed three times with 100 mM sodium phosphate (pH 8.0), 600 mM sodium chloride, and 0.02% Tween 20 supplemented with 10 mM imidazole and then eluted with 50 mM sodium phosphate (pH 8.0), 300 mM sodium chloride, 0.01% Tween 20, and 300 mM imidazole. Fractions were subjected to SDS-PAGE.
