# Supplementary figures and images for: PorZ, an Essential Component of the Type IX Secretion System of Porphyromonas gingivalis, Delivers Anionic Lipopolysaccharide to the PorU Sortase for Transpeptidase Processing of T9SS Cargo Proteins
Source: mBio. 2021 Feb 23;12(1):e02262-20. doi: 10.1128/mBio.02262-20 (PMC8545088; doi:10.1128/mBio.02262-20)

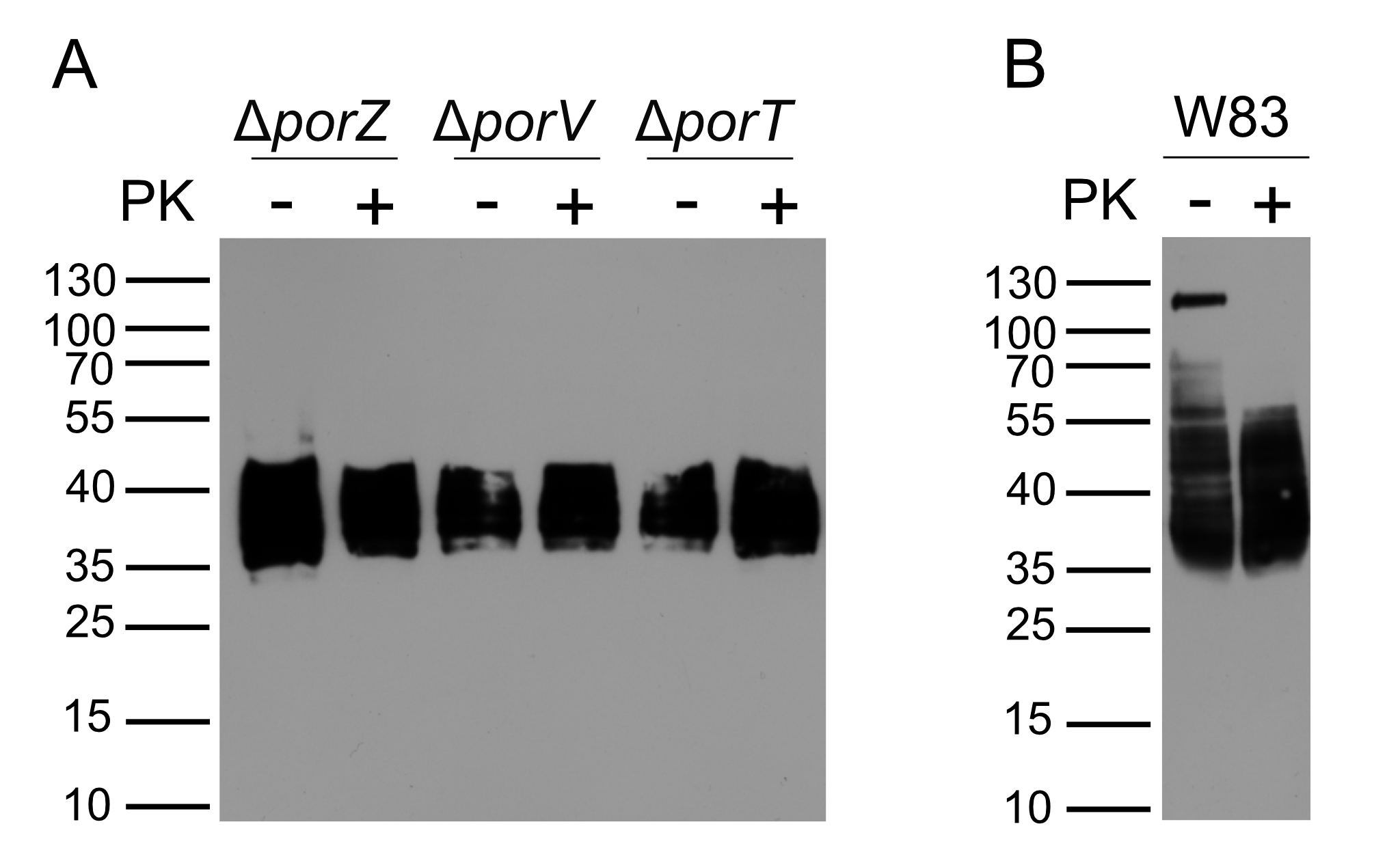

Supplement: FIG S1 [file mbio.02262-20-sf001.tif]

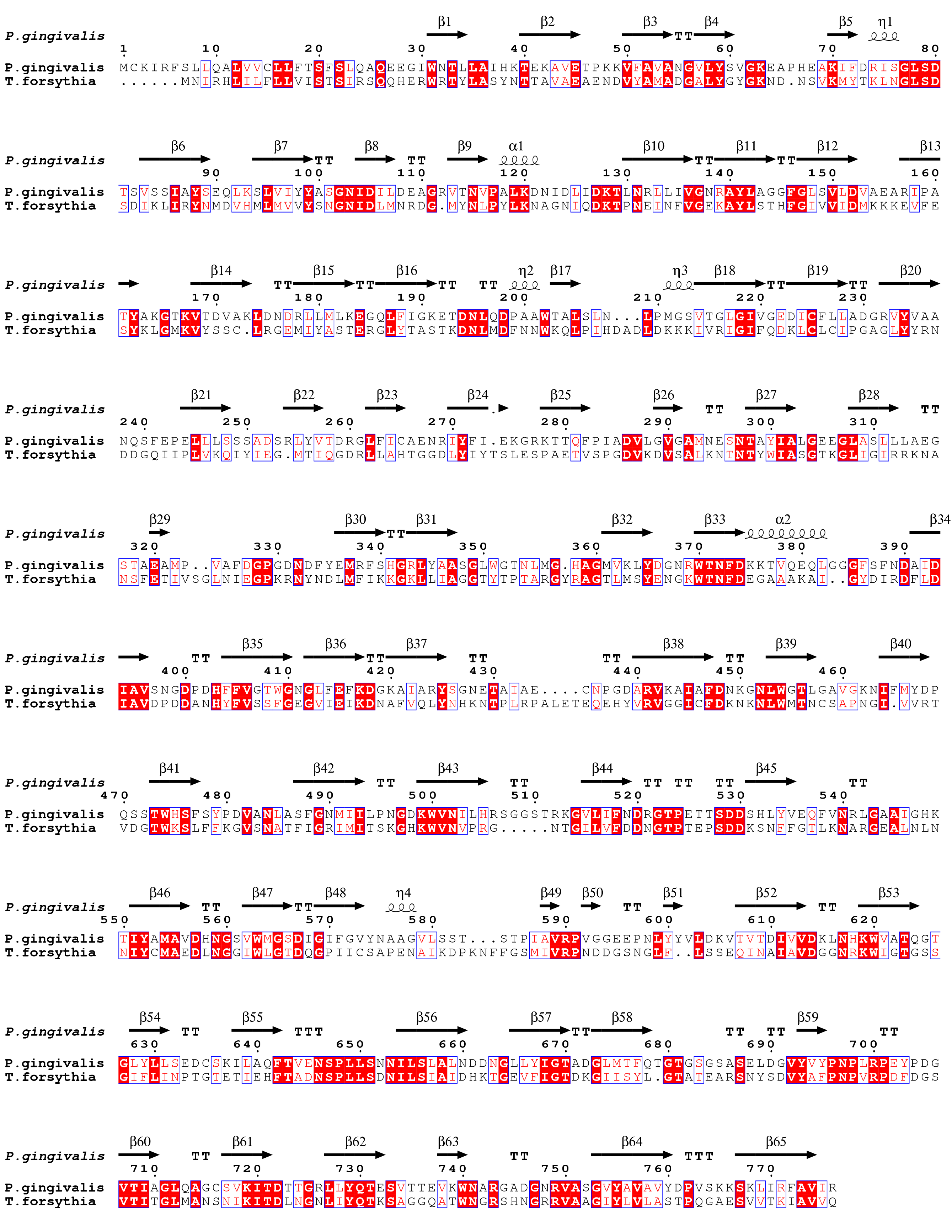

Supplement: FIG S2A [file mbio.02262-20-sf002a.tif]

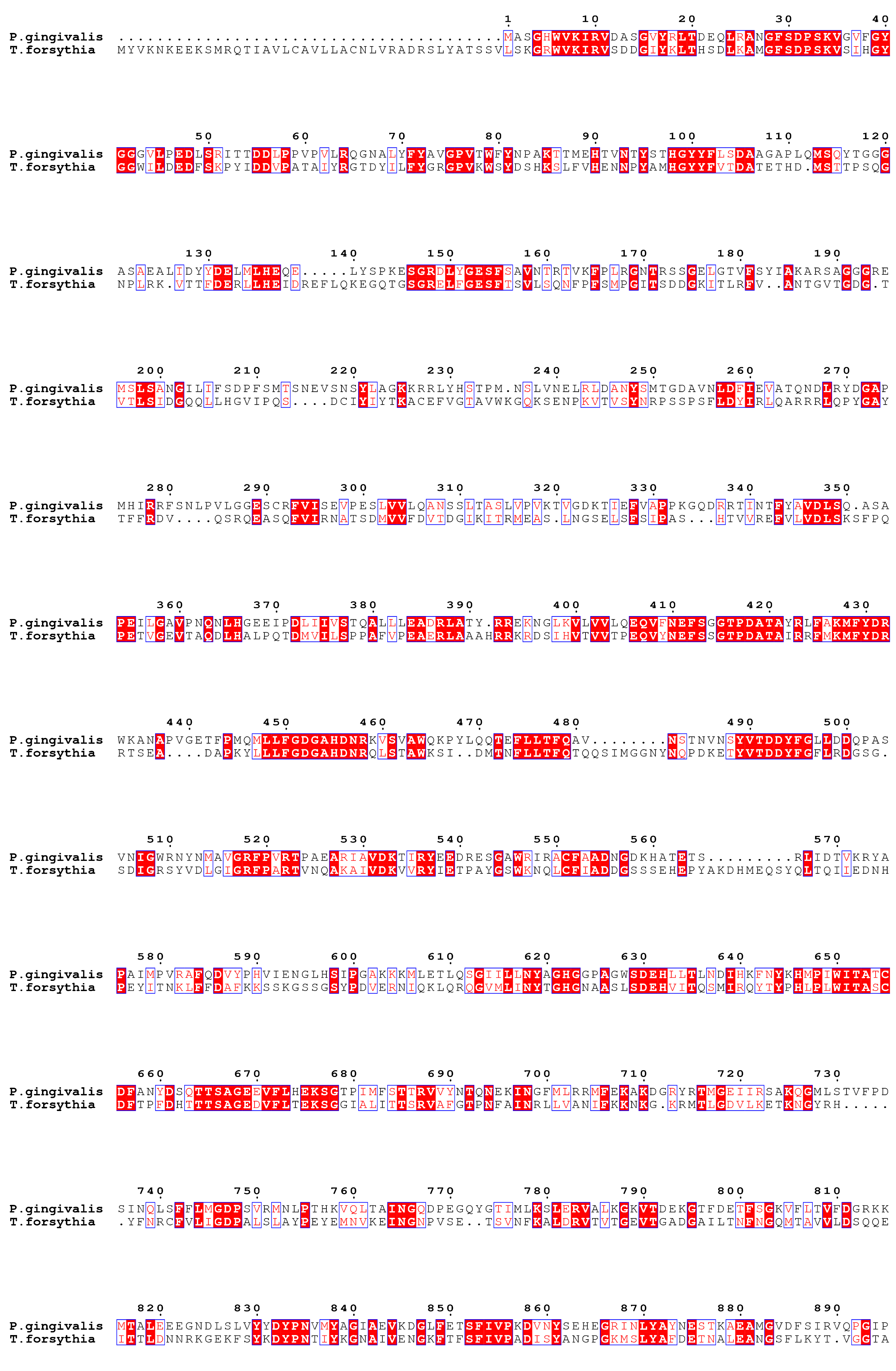

Supplement: FIG S2B1 [file mbio.02262-20-sf002b1.tif]

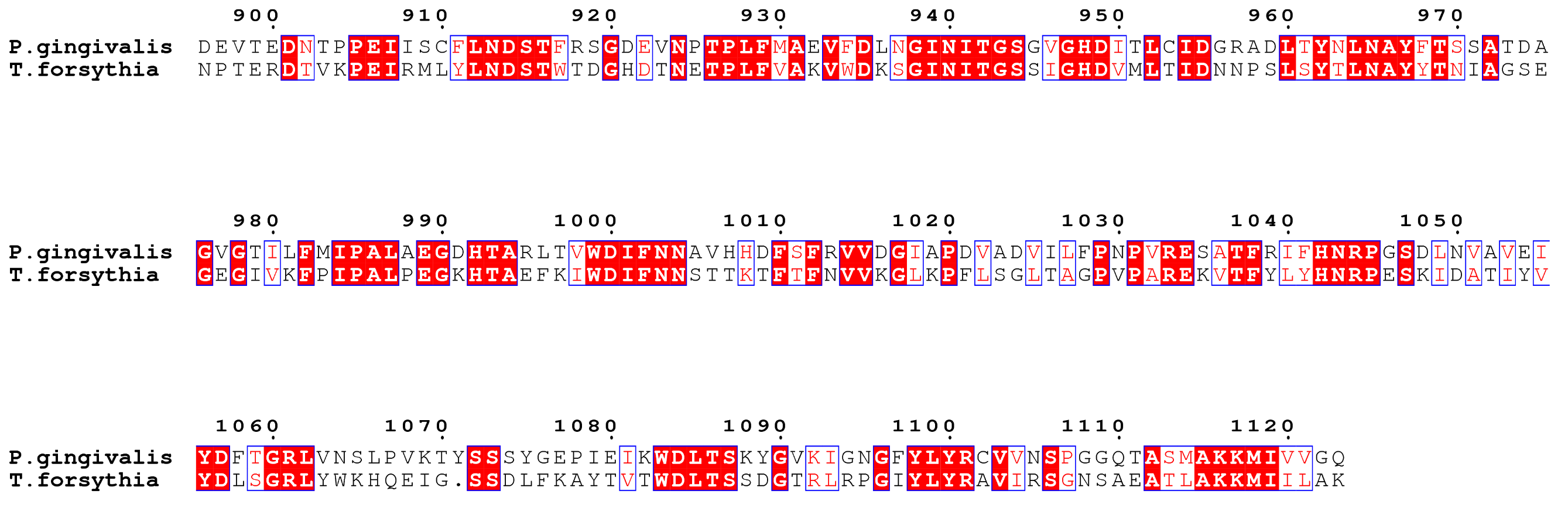

Supplement: FIG S2B2 [file mbio.02262-20-sf002b2.tif]

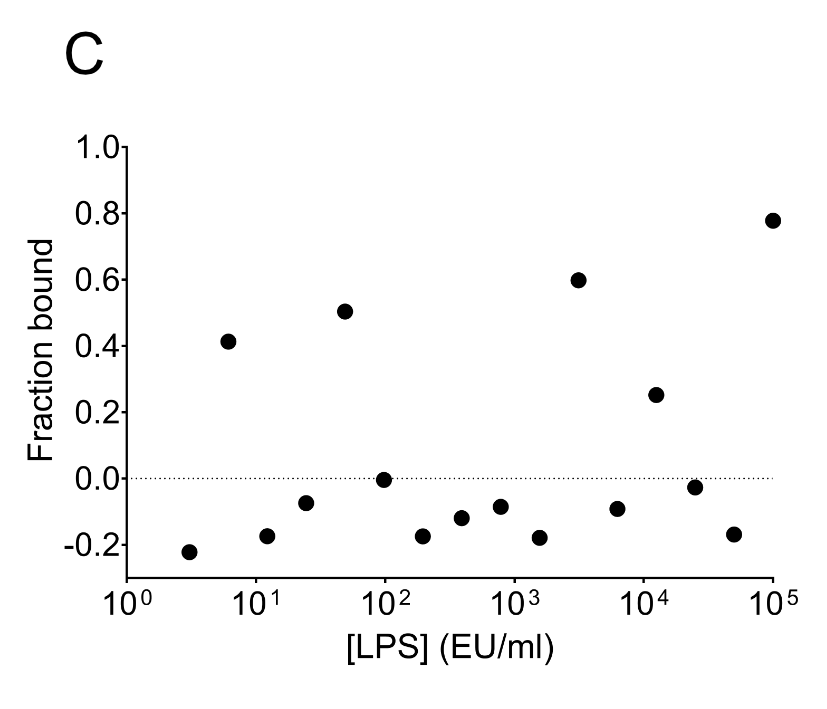

Supplement: FIG S2C [file mbio.02262-20-sf002c.tif]
