# Supplementary material for: PorZ, an Essential Component of the Type IX Secretion System of Porphyromonas gingivalis, Delivers Anionic Lipopolysaccharide to the PorU Sortase for Transpeptidase Processing of T9SS Cargo Proteins
Source: mBio. 2021 Feb 23;12(1):e02262-20. doi: 10.1128/mBio.02262-20 (PMC8545088; doi:10.1128/mBio.02262-20)
Supplement: TABLE S1 [file mbio.02262-20-st001.docx]

| **S1 Table *P. gingivalis* strains used in this study** | | |
| --- | --- | --- |
| **Strain** | **Relevant genotype** | **Source** |
| W83 | Wild-type | Reference strain |
| HG66 | Wild-type | Reference strain |
| Δ*porZ* | *porZ* (NCBI: PG_RS07070; old locus *pg1604*) (Em^r^) | [32] |
| Δ*porT* | *porT* (NCBI: PG_RS03295; old locus *pg0751*) (Tc^r^) | [34] |
| Δ*porV* | *porV* (NCBI: PG_RS00125; old locus *pg0027*) (Em^r^) | This study |
| Δ*pg0129* | *pg0129* (NCBI: PG_RS00600; old locus *pg0129*) (Em^r^) | [36] |
| Δ*pg1142* | *pg1142* (NCBI: PG_RS05080; old locus *pg1142*) (Em^r^) | [36] |
