# Supplementary material for: PorZ, an Essential Component of the Type IX Secretion System of Porphyromonas gingivalis, Delivers Anionic Lipopolysaccharide to the PorU Sortase for Transpeptidase Processing of T9SS Cargo Proteins
Source: mBio. 2021 Feb 23;12(1):e02262-20. doi: 10.1128/mBio.02262-20 (PMC8545088; doi:10.1128/mBio.02262-20)
Supplement: TABLE S2 [file mbio.02262-20-st002.docx]

| **S2 Table *E. coli* strains and plasmids used in this study** | | |
| --- | --- | --- |
| **Strain** | **Relevant genotype** | **Source** |
| NEB® 5-alpha | fhuA2 Δ(argF-lacZ)U169 phoA glnV44 Φ80 Δ(lacZ)M15 gyrA96 recA1 relA1 endA1 thi-1 hsdR17 | New England Biolabs |
| TOP10 | F- mcrA Δ ( mrr-hsdRMS-mcrBC) Φ 80lacZΔ M15 Δ lacX74 recA1 araD139 Δ ( araleu)7697 galU galK rpsL (StrR) endA1 nupG | Invitrogen |
| BL21(DE3) | fhuA2 [lon] ompT gal (λ DE3) [dcm] ∆hsdS λ DE3 = λ sBamHIo ∆EcoRI-B int::(lacI::PlacUV5::T7 gene1) i21 ∆nin5 | Invitrogen |
| **Plasmid** | **Relevant features** | **Source** |
| pUC19 | *E. coli* cloning vector; Ap^r^ | Thermo Scientific |
| pVA2198 | *E. coli-Bacteroides* shuttle vector*,* source of ermF-ermAM cassette; Sp^r^ | [35] |
| pETDuet-1 | *E. coli* expression vector | Novagen |
| P23AeB-B | Plasmid for porV deletion mutagenesis, derivative of pUC19 | This study |
| pETDuet-1/PorU | Plasmid for PorU purification from *E. coli*, derivative of pETDuet-1 | This study |
| pETDuet-1/PorU_Tf | Plasmid for PorU (*T. forsythia*) purification from *E. coli*, derivative of pETDuet-1 | This study  GenScript |
| pGEX-6P-1/PorZ_Tf | Plasmid for PorZ (*T. forsythia*) purification from *E. coli*, derivative of pGEX-6P-1 | This study  GenScript |
