# Supplementary material for: PorZ, an Essential Component of the Type IX Secretion System of Porphyromonas gingivalis, Delivers Anionic Lipopolysaccharide to the PorU Sortase for Transpeptidase Processing of T9SS Cargo Proteins
Source: mBio. 2021 Feb 23;12(1):e02262-20. doi: 10.1128/mBio.02262-20 (PMC8545088; doi:10.1128/mBio.02262-20)
Supplement: TABLE S3 [file mbio.02262-20-st003.docx]

| **S3 Table** **Primers used in this study** | |
| --- | --- |
| **Primer name** | **Sequence (5’->3’)** |
| **pPorV/pUC19/Erm plasmid for deletion of porV** | |
| **PG23FrBXbaIF** | CTATCTAGAATCAAGATGAAGAAGCCTTTTCG |
| **PG23FrBHind3R** | GTCAAGCTTTTTTCGGTTAGAAGGGCTATGC |
| **PG23FrASacIF** | CATGAGCTCGACAGCTTTGGAAGAGGAG |
| **PG23FrASmaR** | GATCCCGGGATTTCGTTCGTTTTGTTTTGTATTC |
| **ermFAMSmaIF** | CATCCCGGGATAGCTTCCGCTATTGCTTTTTTGC |
| **ermFAMXbaIR** | GTGTCTAGATCTAGAGGATCCCCGAAGCTG |
| **Primers for recombinant PorU production from pETDuet-1/PorU plasmid** | |
| **PG26-F** | TGGACTGCAGCAACGAGCTATGGGGAAGACGG |
| **PG26-R** | CTGGCTCGAGCTATTGTCCTACCACGATCATTTTCTTGG |
